# Determinants of vegetable food choice in families with limited food budgets in England: a focus group study to inform vegetable promotion programmes

**DOI:** 10.1017/jns.2025.10016

**Published:** 2025-06-13

**Authors:** Carol A. Williams, Martina Gregori, Nigel Sherriff

**Affiliations:** 1 School of Education, Sport & Health Sciences, University of Brighton, Brighton & Hove, UK; 2 Public Health Team, Brighton & Hove City Council, Brighton & Hove, UK; 3 Centre for Transforming Sexuality & Gender (CTSG), University of Brighton, Brighton & Hove, UK

**Keywords:** Food choice, Inequalities, Limited food budgets, Vegetables

## Abstract

Vegetable consumption in many countries is less than recommended and even lower in low-income households. This study explored the determinants of current vegetable food choice in households with limited food budgets to inform the implementation of a national vegetable promotion programme. Five focus groups and one individual interview were conducted with twenty-nine parents who self-identified as ‘shopping on a budget’ in an area of multiple deprivation in the southeast of England. Transcripts of audio recordings were coded in NVivo and analysed using inductive thematic analysis. Four main themes which shaped the range of vegetables brought into the home were identified: (1) attributes of vegetables, (2) attributes of parents including their vegetable norms, knowledge and skills (veg-literacy), and interest and opportunity to invest time and effort in vegetables, (3) family food dynamics, and (4) influence of retailers. Overarching this was parents’ capacity to absorb the risk of wasting food, money, time, and effort on vegetables and damaging trust in the parent–child food relationship. The data suggest there is a common set of ‘core vegetables’, which are routinely bought. When money is tight, parents only buy vegetables they know their children will eat and are generally not persuaded to buy ‘off-list’ in response to price discounts or promotions. Cost is not always the main barrier to increased vegetable purchase. To avoid unintentionally widening dietary inequalities, supply-side interventions to promote vegetable consumption need to be designed alongside targeted actions that enhance the capacity of low-income households to respond.

## Introduction

Eating more than 400 g of fruit and vegetables has been a key component of global population dietary guidance for the prevention of chronic diseases for more than 30 years.^(1)^ However, average intakes remain below the recommendation^(2–4)^ and have a clear social gradient.^(5–8)^

In the UK, the 400 g goal was pragmatically translated into advice to consume at least five 80 g portions of different fruit and vegetables a day, and the government’s nutrition task force produced guidance on what was included.^(9)^ Globally, recommendations are similar with portion sizes between 80 and 100 g, although there is some cultural and contextual variation on the inclusion of vegetables such as potatoes and sweetcorn.^(4,10)^ WHO’s 400 g/day goal is a ‘lower limit population goal’, and the public-facing message has always been to eat ‘at least’ *5-a-day*. The evidence in favour of plant-based diets with high proportions of fruit and vegetables continues to grow,^(11)^ with some studies suggesting benefits of 600–800 g/day.^(12,13)^ The 2019 Lancet Eat commission looked at healthy diets in the context of planetary health and included 300 g of vegetables and 200 g of fruit in its healthy reference diet,^(14)^ equivalent to around six portions. Several high-income countries now recommend at least 7 portions or 500 g or more, with specific emphasis that half or more should come from vegetables, including Australia, Denmark, Germany, Finland, and Iceland.^(15,16)^ The UK’s 2016 national food-based dietary guideline, the Eatwell Guide, represents recommended intakes of approximately 550 g/day, equivalent to around four portions of vegetables and three of fruit.^(17,18)^

The launch of a ‘*5-a-day’* campaign in the UK in 2003 raised the profile of the advice, and it is widely known.^(19)^ Survey data show a steady increase in adult ‘*5-a-day’* compliers from 24% in 2001 to 34% in 2022; however, there has been no change in the zero/less than one portion consumers (11%), and differences between income groups remain (Table 1).^(20)^ As public health emphasis on the benefits of vegetables and plant-based diets has strengthened, the social gradient between high and low vegetable consumers risks increasing, exacerbated by the cost-of-living crisis.^(21,22)^

**Table 1.** Comparison of fruit and vegetable consumption by the highest and lowest index multiple deprivation (IMD). *Data source*: Health Survey for England, 2022, Part 1(20)

	Adults 16+ years		Children 5–15 years	
	IMD quintile		IMD quintile	
Portions per day	Least deprived	Most deprived	Least deprived	Most deprived
% None (*zero consumers*)	4	12	6	12
% Less than one portion	3	5	6	3
% 5 or more portions (*compliers*)	34	23	25	17
Mean portions	4.4	3.3	3.5	3
Median portions	3.7	2.7	3	2.7

Many studies identify the price of vegetables as a key barrier for households on a low income,^(23–25)^ and there is a widely documented association between low income and low purchase.^(26)^ However, price or availability are not the only barriers or necessarily the most impactful. Other studies have noted a correlation of intakes with education independently of income.^(5,26)^ There is some evidence that those who eat the least vegetables perceive them as being less available^(27)^ or have low knowledge or familiarity with vegetables^(28)^ compared with higher consumers.

Understanding current practice is essential to designing effective public health interventions,^(29)^ and from a social justice perspective, this includes understanding practice in the sectors of society known to face the biggest challenges.^(30)^ In common with other high-income countries, the majority of food purchases in the UK are from supermarkets. This study sought to better understand the determinants of the current vegetable food choice of parents with limited funds for grocery shopping, in an area of multiple deprivation in the Southeast of England. It was designed to assist local implementers of a national vegetable promotion programme, the ‘Peas Please’ initiative, which worked with retailers and other partners across the food system with the aim of making it easier for everyone to eat more vegetables.^(21)^

## Methods

### Design

Much of the discourse examining dietary practices in low-income groups takes a deficit model such as examining barriers to eating more healthily. In contrast, this study used an assets-based framing, which starts with understanding current vegetable choices. Asset-based perspectives seek to understand existing positive health practices so that health promotion interventions can be designed with an appreciation of the existing strengths of individuals or communities.^(31)^ A qualitative study was conducted using focus group discussions (FGD) to explore how parents choose, buy, store, cook, serve, and eat the vegetables they do. Participatory techniques were incorporated to elicit parents’ views and encourage the development of shared narratives. A short, structured questionnaire was used to collect demographic information. This study was conducted according to the guidelines laid down in the Declaration of Helsinki, and all procedures involving research study participants were approved by the University of Brighton’s School of Health Sciences Research and Ethics panel 13042018. Written informed consent was obtained from all participants.

### Participants and recruitment

The study recruited parents or carers with children under 18 years regularly living with them, self-identified as ‘shopping on a budget’, and shopped in the local discount supermarket in one of the 10% most deprived neighbourhoods in the UK in the Southeast of England. Eligible participants had to understand and speak English. Recruitment took place between April and June 2018 using posters, flyers, and active on-site recruitment by the research team in the supermarket, at primary and nursery school gates at collection times, and in a local community youth centre. All participants received information about the study before providing informed consent and received a £20 shopping voucher as recompense for their time.

### Data collection and analysis

FGDs took approximately 1.5 hrs and were held in quiet private rooms at three local community venues selected for being neutral non-official premises in different parts of the neighbourhood. The moderator was author 1 and facilitator author 2, who are both trained in qualitative research techniques. Each FGD was audio recorded and began with icebreaker questions about children’s favourite meals. One of every fresh vegetable and salad item on sale in the local discount supermarket on the day of the FGD (approximately forty items) and a selection of common canned vegetables, were laid out on a central table, alongside a supermarket basket. Participants were asked to identify which vegetables they ate regularly and would be annoyed if they forgot to buy when doing a food shop. Each vegetable was discussed in depth and placed into the basket. The discussion then moved onto vegetables used less often, rarely, or never, using three A4-size colour photographs of vegetables in the freezer cabinet and in-store displays as prompts as appropriate. Specific probes about cooking and preparation, special offers, and frozen vegetables were raised as needed. The vegetables in the room helped create a friendly, exploratory, and non-judgemental environment. The FGD topic guide was used flexibly to allow participants’ interests and interactions to steer the discussion.

Data were stored securely using password-protected General Data Protection Regulation-compliant cloud storage. Participants were allocated unique and anonymous participant numbers. Recordings were transcribed and checked by members of the research team, with one exception, which was transcribed by an external university-approved supplier. Thematic analysis was used to inductively code and analyse the data using a six-stage process as outlined in Braun and Clarke.^(32)^ Transcripts were iteratively read and coded using qualitative data software (NVivo) to support the process. Initially 80+ codes were generated; these were reviewed, refined, and clustered into themes and sub-themes through recursive re-reading, mapping, and defining themes. Themes were generated by author 1 and reviewed by authors 2 and 3 in relation to the general pool of codes and the entire data set.

## Results

Five focus groups with four to eight participants and one in-depth interview (due to non-attendance) were conducted with a total of twenty-nine participants. After sequential analysis of the first five FGDs, no new themes emerged, and recruitment was stopped. Study participants were mostly white British, aged between 25 and 44 years, unemployed or in part-time employment, and with qualifications of lower secondary education (General Certificate of Secondary Education) or below (Table 2). Analysis of postcode data by index of multiple deprivation (IMD) indicated that 76% of participants lived in areas in the lowest quintile of deprivation. Of the 29 parents or carers in the study, 14 had one child (48%), 6 two children (21%), and 9 three children (31%) under 18 years regularly living with them. The term ‘parent’ or ‘participant’ is used throughout this paper to denote the main carer responsible for decision-making around family food. Where terms have been generated during the analysis to summarise participants’ conceptualisations or perceptions, these are in italics, and italics and quotation marks when arising from verbatim comments.

**Table 2.** Demographic characteristics of participants

Total sample	N = 29		
Gender		Educational qualifications	
Male	4	None	7
Female	25	General Certificate of Secondary Education (level 2 ISCE)	8
Age		Advanced levels/diploma (level 3 ISCE)	4
20–24 years	2	Undergraduate	2
25–34 years	9	Professional qualification	1
35–44 years	6	Not known	7
45–54 years	3	Health	
55–64 years	0	Has ongoing health problem or disability	9
65+ years	2	Employment status	
Ethnicity		Employed full time	0
White British	18	Employed part time	9
Irish	1	Unemployed	14
White Asian	1	Retired	2
Indian	1	Not known	4
Religion		Index of multiple deprivation quintile based on postcode data	
None	15		
Christianity	5	Most deprived: Band 1	22 (76%)
Muslim	1	Band 2	5
Marital status		Band 3	1
Married/cohabiting	10	Band 4	1
Partner (not living together)	2	Least deprived: Band 5	0
Single	11		
Separated/divorced	5		
Widow/er	1		

### Identified themes

The findings have been clustered into four broad emergent themes reflecting distinct areas for consideration when designing vegetable promotion interventions: attributes of vegetables, attributes of parents, family food dynamics, and influence of the retailer (Table 3). Representative examples of participant quotes for each sub-theme are shown in Tables 4–7, identified as M, F, GM, or GF for mother, father, grandmother, and grandfather, respectively. The first digit indicates the focus group; thus, M15 is mother, number 5 in focus group 1, and MI for the individual interview.

**Table 3.** Summary of themes and sub-themes. Verbatim terms from the focus group discussion in italics

1. Attributes of Vegetables: non-modifiable properties that determine intakes. a. ‘*Core veg*’ — routinely purchased by most parents. b. ‘*Love/hate veg*’ — never bought or often bought. c. Frozen veg
2. Attribute of Parents: parents as gatekeepers of selection which children are exposed to at home. a. Habits, preferences, and norms. b. Veg-literacy — parents’ apparent familiarity, skills, knowledge, and self-efficacy with vegetables. c. Interest and opportunity to cook veg
3. Family food dynamics: how parents respond to children’s vegetable food preferences. a. Picky or contrary eating b. Shrinking range of vegetables c. Exposure and modelling of vegetable eating to children d. *‘Sneaking it in’* — disguising vegetables in children’s meals.
4. Influence of retailer. a. Pack size and food waste b. Response to price promotions

**Table 4.** Theme: Attributes of vegetables, sub-themes, and illustrative quotes

Theme: Attributes of vegetables	
a. Core veg	*‘You can eat them raw; you don’t have to cook them’. (M12)* *‘They are quite convenient for snacks and lunch boxes’. (M15)* *‘They’re colourful and kids will eat ‘em’. (F13)* *‘Of all the kids that I know cucumber is the one staple that they will all pretty much eat’. (M44) ‘It’s a safe vegetable’*. *(M43)* *‘cos they’re cost effective I think, the cost of them is a lot lower than some other vegetables’. (M23)*
b. Love-hate vegetables	*‘Mushrooms are one of those things you either love them or hate them’. (M23)* *‘See I love spinach. For me it would be a catastrophe to forget* (to buy it*) but for my kids it’s too slimey’. (M44)*
c. Frozen vegetables	*‘I don’t do no frozen’. (M17)* *‘The only thing we’ve got frozen is onions’. (F13)* *‘I literally only buy it in case we’re running late for dinner or whatever’. (M52)* *‘The only one is frozen peas, I think they taste better than tinned’. (M51)* *‘It takes away the goodness as well when you freeze vegetables’. (M24)*

**Table 5.** Theme: Attributes of parents, sub-themes, and illustrative quotes

Theme: Attributes of parents	
a. Habits, preferences, and norms	*‘You tend to go week by week’. (M17) ‘Have the same things’. (M12) ‘You have the same’. (M16)* *‘I am one of those really naughty parents; I just assume the kids wouldn’t like it if I don’t like it’. (MI)* *‘I’ve not tried it and I don’t want to’. (M21)* (Radish) (strongly*)* *‘I would cook it if I was cooking a roast, but it’s not the norm’. (M24)* (cauliflower) *‘I don’t have them a lot, they are quite fancy when they are on a plate’. (M21)* (green beans) *‘Yeah, like more vegetarians’ type food’. (M24)* (aubergine)
*b. Veg literacy*	*‘Isn’t it white, I thought the spinach could come in white?’ (M21)* *‘I’ve only ever had it ‘cos someone else has cooked it for me and I was really reluctant to try it, but it is actually really nice, I had it roasted with chicken. But I’ve never bought it to do it myself’. (M24)* (beetroot) *‘I went away once and I was with a lot of vegans, and they put courgette in a lasagne and I would never ever think of doing it and do you know what, it was lovely and I think there was aubergines in there as well and I’ve never would have thought of putting that in’. (M17)* ‘*It’s all on upbringing, so my mum doesn’t like veg anyways, obviously she would give us veg, but she wouldn’t really give us things outside the box* (the basket of core veg on table) *so I wouldn’t really know what do to’. (M21)*
*c. Interest and opportunity to cook veg*	*‘I cook a lot less since I’ve had a child. For a single parent, working and having a child… I used to use a lot more of this sort of veg when I used to cook more frequently’. (M12)* *‘I’m not cooking three meals a day for love nor money or the tea in China, I just can’t, I haven’t got time to cook three meals a day’. (F53)*

**Table 6.** Theme: Family food dynamics, sub-themes, and illustrative quotes

Theme: Family food dynamics	
a. Fussy or contrary eating	*‘I thought my son was fussy. I’ve been talking to other people; I realise that it’s just how kids are’. (M15)* *‘All different, yeah, I can’t really just go home and cook one meal’. ‘Yeah, the older one if I give her a plate of vegetables she will sit there and eat it, but my youngest one she would go, “I ain’t eating that”, and then go and put it in the bin’. (M51)* *‘I’ve got a child that’ll go to my cousins and eat every single thing that’s put on her plate and come home and swear blind that she doesn’t like it’. (M55)* *‘She won’t eat the sweetcorn from the tin but will happily eat corn on the cob from KFC or if I buy corn on the cob, I’m thinking it’s the same baby’. (M21)*
*b. Shrinking range of veg.*	*‘As a* (young) *child, before she got what she perceives as good or bad* (foods*) she would eat all of this on the table, but now she’s quite fussy’. (M18)* *‘She’s very fussy. She used to eat anything and everything when she was little. But as she got older, she eats too much of one flavour and then goes off it. Basically, she’s worked her way through all the good foods and all the fruit and vegetables, so she doesn’t eat them anymore’. (M43)*
c. Exposure and modelling of veg eating to children	*‘The youngest sees that I don’t eat them* (vegetables)*. “So, it’s not on your plate so why should I eat it?”’ (M51)* *‘A very good way I got round it was making them have school dinners because then they’re eating a proper homemade meal and then coming home saying ‘oh mum I liked it’. (M11)* *‘My son likes tomatoes a lot, like every lunch he will have tomatoes. He didn’t like them then he had them at school and ever since he’s been obsessed with tomatoes’. (M23)* *‘They made a salad at school. They were all having fun making it and he came home and that spurred him on to want to try salad a bit more’. (M44)*
d. Sneaking it in	*‘Sneaking it in is better cos if they see it they will say ‘I don’t want that’, but otherwise if you just give them without them knowing it they eat it. They won’t even know’. (M22)* *‘See my son wouldn’t like that. He would feel like I was tricking him. He would rather know what’s in the food and then try it. He’s seven’. (M23)*

**Table 7.** Theme: Influence of retailers, sub-themes, and illustrative quotes

Theme: Influence of retailers	
a. Pack size and waste	*‘Because I’m the only one eating it, so unless I want to eat spinach every night, I don’t buy spinach cos it comes in this big plastic bag’. (F33)* *‘I just remember as a kid myself all the fruit and veg came in brown paper bags…and you buy what you need…, whereas here you are kinda forced to buy a huge bag’. (M16)* *‘Smaller packs, not for all households, but for small households, if you got a whole family of five, they can eat a whole broccoli but if you got a family of two’. (M23)*
e. Response to price promotions	*‘It depends what they are selling (*on special offer)*, if it’s the tomatoes and lettuce you’re going to grab them’. (M11)* *‘No, if it’s something that we do want to buy and it’s on offer then [yes], but otherwise’. (M22) ‘I wouldn’t buy if I wouldn’t normally buy it’. (M24)* *‘I don’t want to spend £2 on this when I don’t think my sons going to eat it, whereas I know if I spend £2 on that (core veg), I know he will eat it. I think that’s where as parents, as adults, we get into that routine of buying stuff that we know our kids are going to eat’. (M15)* *‘Yeah. I don’t want wastage because if we can’t hardly afford to eat, I don’t want anything wasted. Even when I have enough money, I can’t stand waste’. (M43)*

### Attributes of vegetables

Participants’ views and experiences of the intrinsic properties of vegetables are presented as a separate theme because these characteristics are not readily modifiable and vegetable promotion interventions need to build on those aspects that favour consumption.

a) ‘*Core veg*’: There was broad agreement on the essentiality of seven or so ‘*core veg*’ that nearly all participants considered as their ‘*veg necessities*’. Participants identified several key characteristics of these ‘*core veg*’, which meant they were routinely purchased: they are ‘*dual use*’, most can be eaten cooked or raw; multipurpose — they can be used in salads, sandwiches, lunchboxes, snacks, or meals; they are easy to prepare; and were constructed as ‘safe’ because children will eat them. The ‘*core veg*’ identified were carrots, tomatoes, cucumber, peppers, onion, broccoli, tinned sweetcorn, and for those that ate them, mushrooms, and salad. These were considered cheaper than other vegetables although this did not necessarily reflect the actual prices; rather, participants spoke of ‘*core veg*’ being used daily and having a high ‘*turn around*’ so they were perceived as ‘*cost effective*’ because they were easy to fully use.

b) ‘*Love/Hate veg*’: Some vegetables provoked a divided response and were strongly disliked, avoided, and never purchased by some participants, while considered easy to eat and regularly bought by others. Reasons for rejection were dislike of a distinctive flavour (beetroot ‘*too earthy*’, celery, radish) or aversion to a ‘*slimy*’ texture (avocados, aubergines, mushrooms). For those parents who disliked these ‘*love/hate*’ vegetables, the sentiment was so strong that it would be a challenge for them to consider buying or cooking them for their children (Table 4).

c) Frozen vegetables: Frozen vegetables rarely came up in the discussion unless prompted by the moderator, and it seemed as though participants felt the need to justify why they used frozen vegetables. Frozen peas were the main exception, but even here, many participants emphasised that they had frozen vegetables as a reserve, for times when they were in a hurry, or to respond to individual children’s preferences. In several of the FGDs, there was discussion and disagreement about whether frozen vegetables were nutritionally better because it was ‘*fresh frozen*’ (M52) or ‘*hadn’t got the goodness*’ (M56). In general, the sentiment was that frozen was not as good as fresh for taste, texture, or nutrition but was widely acknowledged as being cheaper.

### Attributes of parents

Parents’ vegetable shopping is shaped by their preferences and ideas about what are ‘normal’ amounts and how they fit into acceptable meal patterns, what they know how to cook and prepare (‘*veg-literacy*’), and their motivation and opportunity to serve vegetables.

a) Habits, preferences, and norms: Participants’ vegetable purchases were strongly influenced by habit; they spoke of going into the shop knowing what they wanted, not looking at promotions, and buying the same vegetables each week. This mainly comprised the ‘*core veg*’, plus other vegetables that ‘*went*’ with planned meals. Many participants referred to buying two or more non-core veg specifically for the traditional Sunday roast dinner and invested in this as an important family meal. These vegetables, such as parsnip, swede, or cauliflower, tended not to be used for other meals and were identified as often being left at the bottom of the fridge and thrown away. Some vegetables were considered the kind that vegetarians or ‘*others*’ would eat, but not them. Parents were aware that vegetables were important, and several gave their children raw ‘*core veg’* as a snack or ‘*starter*’. When asked about the place of vegetables in other common meals, such as spaghetti bolognaise or pizza, some parents explained that ‘T*here is already veg in there*’ (M23) (tomato and onion), and it was unnecessary to serve additional vegetables on the side. There were some exceptions to this; one parent spoke of serving coleslaw or other ready-made salads alongside so that ‘*They’ve got a plate of food not just two slices of pizza. It makes it a dinner*’, pointing out that, ‘*If you go to Pizza Hut or somewhere you get a salad with it*’ (MI).

b) *Veg-literacy*: In every FGD, there were one or two vegetables which few participants recognised (such as pak choi or celeriac) and some which several participants were uncertain how to use and only ate when other people cooked them (such as aubergine). In every FGD, there were also one or two participants who were unfamiliar with many of the vegetables (Table 5). The combination of exposure and familiarity with vegetables (‘veg knowledge’) combined with skills to select, prepare, and cook vegetables and the confidence to put this into practice (self-efficacy) and serve vegetables is what we have termed *veg-literacy*. The discussions suggested that initially, *veg-literacy* is shaped by parents’ exposure to different vegetables and cooking skills in childhood. Participants spoke of learning how to cook certain vegetables from their mothers or at school in home economics lessons. A few participants talked of knowing how to cook a wider range of vegetables than their parents, for example, through working in the catering trade or living in shared accommodation before having children. *Veg-literacy* seemed to be lower in younger parents and those who reported having minimal exposure to vegetable diversity in their own remembered childhood. One participant explained that when she became pregnant, she was young and still quite fussy about what she would eat, so she did not know what to do with a lot of vegetables; now, as a parent, she felt hampered by this. Others spoke of needing to see and taste an unknown vegetable before having the confidence to buy. Participants who seemed more *veg-literate* used a wider range of vegetables and cooking methods and had effective techniques for avoiding waste, using leftovers or vegetables that were starting to go off. They tended to be less likely to comment that vegetables were expensive.

c) Interest and opportunity to cook veg: This sub-theme brings together parents’ level of interest and practical opportunity for engagement in giving time, money, and effort to preparing and cooking vegetables, and getting the rest of the family to eat them. Although linked with *veg-literacy*, some of those who seemed most *veg-literate* and motivated were juggling many different professional and caring roles and complained that they had less opportunity to cook vegetables than they would have liked. Some parents clearly enjoyed cooking and were happy to spend time in the kitchen as a means of relaxation. For others, it was a chore or had become a chore that they struggled to fit in amongst other aspects of family life, particularly for single parents. Time was a recurrent limitation. Most parents recognised vegetables as an important part of their children’s diets and were therefore quite motivated to do things that would encourage their children to eat vegetables when they had time and enjoyed sharing their experiences. This ranged from using spiralisers, putting veg into smoothies, and involving children in food preparation.

### Family food dynamics

This theme looks at the influence of interactions between parents and children around vegetable preferences and rejection. Some households accommodated children’s preferences by cooking different meals or different vegetables for individual family members, which meant the ‘picky’ child tended to have the same narrow range of ‘favoured’ or ‘accepted’ vegetables for most meals. In others, children’s preferences shaped and limited what was purchased and served for the entire household.

a) Picky or contrary eating: Most parents in the study had experience of children being picky about eating vegetables and expressed their frustration and exasperation (Table 6). Often, it was one child who was perceived as fussy while the others were good eaters. Some parents described behaviours that were consistent with general definitions of fussy eating: an unwillingness to eat familiar foods, try new foods, and strong food preferences.^(33)^ Others described behaviour that was constructed as contrary, for example, ‘*eating cauliflower at Nan’s, but not at home*’ (M55). Few seemed prepared to consider that the rejection of a vegetable at home might be because of the way it was cooked or served or the food that it was cooked with.

b) Shrinking range of vegetables: There seemed to be an acceptance and expectation that children would go through a phase when the range of vegetables they would eat shrinks and that this might last throughout the school years. Some spoke of children liking all sorts of vegetables until ‘*they’re old enough to decide for themselves, then they wouldn’t go near it*’ (M44). Parents clearly framed this as a phase, citing how children had ‘*gone off*’ rather than never eaten the vegetable. Parents with older children readily shared experiences that they had found useful in restoring the range of vegetables their children would eat, and at what age to expect an improvement. One parent spoke of encouraging her children to ‘*“try it again because as you get older your taste buds change” and I found that actually works quite a lot*’ (M16).

c) Exposure and modelling of veg eating to children: Parents of children who were ‘*good veg eaters*’ were regarded as fortunate, and other parents were curious about how this had been achieved. Some referred to the benefits of introducing different vegetables at an early age, when children tend to be more accepting. Parents reflected on their own behaviours; those who did not like vegetables themselves, or eat much, were not surprised that their children were unenthusiastic about the vegetables they served. Many spoke of the valuable influence of exposure to vegetables at school, noting that in the school/nursery environment, children were prepared to eat things which they would not eat at home, referring to the influence of peers and copying what others did. However, this was not always positive; a few spoke of their children going to school and copying the food rejection of others.

d) ‘*Sneaking it in*’: Opinions on disguising vegetables so that children will eat them were divided. Parents fully articulated the pros and cons of ‘sneaking’ vegetables into dishes, on the one hand, being an effective method of ensuring children ate their vegetables, but on the other, failing to educate and expand the conscious range of vegetables which children would eat and risking child–parent trust around meals. Examples of techniques for hiding vegetables in meals were more likely to be brought up by parents who were confident of their cooking methods and techniques. Awareness of the importance of diversity was low; a few acknowledged that their children ate ‘*A limited range*’ (M23). Overall, parents seemed happy (or relieved) if their child ate regularly from the ‘core veg’, then this was enough.

### Influence of Retailers

a) Pack size and food waste: Most vegetables and salad sold in the supermarket are in prepacks, so quantities purchased are decided by the retailer, not the customer. Being required to buy more than was needed was a barrier to purchase, particularly for small families or households not eating many vegetables (Table 7). The risk of waste deterred some parents from buying heads of broccoli or fresh cauliflower or bags of vegetables/salad for themselves, which the children did not eat. There was general support for the idea of smaller packs, provided these were not disproportionately more expensive.

b) Response to price promotions: The response of most participants to questions around price promotions, discounts, or money-off vouchers for vegetables indicated how little flexibility most of the participants had to respond to such incentives. Generally, participants were pleased when special offers were on vegetables, which they usually bought (‘core veg’), but the discount did not make them buy more; instead, any saving was used to offset costs on other parts of the grocery bill. They were generally reluctant to buy anything that was not what they usually bought (‘off-list’). Several spoke of responding to special offers on fruit, but with vegetables, they were more likely to be ‘*planning your meals for the week to a budget, so you have got in mind about what (*veg*) is needed*’ (M44). The overriding message was that when money is tight, parents only buy what they know their kids will eat.

## Discussion

**Fig. 1.**
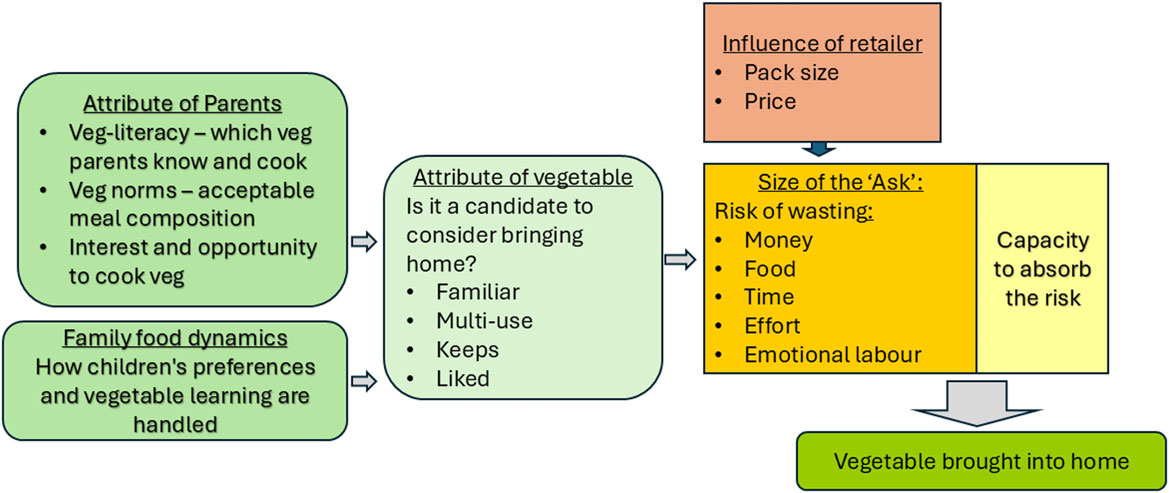
Theoretical schema illustrating how determinants of vegetable food choice influence vegetable shopping decisions of parents who ‘shop on a budget’.

Our research sought to better understand how parents with limited funds for grocery shopping make decisions around buying vegetables from a supermarket and point to possible entry points for vegetable promotion interventions as part of wider public health initiatives. Our findings reveal the complex interplay of determinants that shape the range of vegetables brought into the home and, thereby, children’s early exposure and normalisation of patterns of vegetable consumption. A theoretical schema outlining how our analysis suggests these factors interact is presented in Fig. 1.

The data suggest that parents who shop on a budget tend to shop for vegetables based on habit, routinely buying a set of ‘*core veg’* or ‘*safe veg*’ they know will get fully used and are liked by their children. Five of the seven *core veg* identified in this study can be eaten without cooking. Unlike most foods on sale in supermarkets, vegetables are usually sold without cooking instructions, may have specific storage requirements, a short storage life, and their price and availability change with the seasons. Serving vegetables can require a higher level of practical food skills and knowledge than serving other foods, particularly for households that mainly use processed foods where meal preparation involves following instructions on packaging.^(34)^

Studies on food literacy and cooking skills note a consistent positive association between cooking skills and vegetable intakes in all population groups.^(35–39)^ Building on existing definitions of food literacy,^(40,41)^ we used the term *veg-literacy* to refer to participants’ apparent familiarity, skills, knowledge, and self-efficacy to select, prepare, cook, serve, and store a range of vegetables, avoiding waste, and reflecting seasonality. Vegetables are one of the main sources of avoidable food waste in the home,^(42)^ so for households with small margins for wasting money on uneaten food, they can be a high-risk item.^(43)^ Low *veg-literacy* exposes parents to a greater risk of waste.

Other authors have also noted risk aversion to wasting food and money as shaping food decisions in low-income families.^(43)^ Our study suggests that risk aversion concerns additionally apply to the opportunity cost of wasting time and effort in preparing and serving vegetables and the risk of food rejection damaging parent–child trust around food and undermining parents’ self-efficacy around their cooking skills. Thus, decision-making is governed by a combination of being able to both afford (time, effort, money) and cope with the consequences of providing more or new vegetables. This seemed to be tempered by parents’ attitudes to the risk and disappointment of food being rejected and their opportunity in terms of time and practical resources to prepare and cook vegetables or meals generally. If parents are intrinsically motivated and interested to get their child to try or retry a new vegetable, they may derive satisfaction from putting their understanding into practice and thus feel less troubled if food is uneaten.

**Fig. 2.**
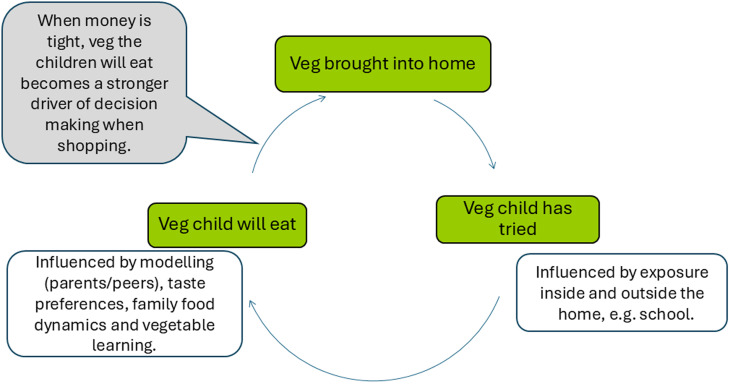
Schema illustrating how children’s exposure to vegetable diversity can shrink in low-budget households.

Parents are gatekeepers of the food and vegetables that are brought into the home.^(44)^ Children’s picky eating around vegetables is widely reported in the literature across all income groups.^(33,45)^ Children need repeated exposure to vegetables in early childhood to learn to accept their often sour or bitter flavours.^(46)^ How parents respond to fussy eating around vegetables influences children’s exposure. Parents in our study, consistent with other studies in low-income families, were strongly motivated to avoid wasting food, tending to only buy vegetables that they knew their children would eat.^(43,47)^ Our findings suggest that if a child is picky about a vegetable, it can get written out of the household’s vegetable shopping habit and not bought again. This can set off a downward spiral whereby children are exposed to, and eat, a shrinking range of vegetables at home (see Fig. 2).

Other studies in low-income households have similarly noted the risk of children having limited exposure to vegetable diversity and missing out on early taste learning experiences.^(43,48,49)^ The relatively limited vegetable diversity is a concern; epidemiological and experimental studies show a clear positive association between the variety of vegetables in the diet and quantities eaten in both adults^(50,51)^ and children^(52,53)^ including in low-income households. Public health nutrition recommendations have consistently recommended eating a variety of fruits and vegetables.^(18)^ Over the past decade, there has been mounting evidence on the importance of the gut microbiome for health and the association between long-term intakes of a variety of fruits and vegetables and a healthful microbiota.^(54–56)^ It is important that vegetable promotion programmes look to increase and monitor both the diversity and amounts of vegetables in the diets of children and other low vegetable consumers.

Parents with higher levels of *veg-literacy* are likely to have more latitude to respond to vegetable promotions, as do larger families where other household members can eat any vegetables children might refuse, avoiding waste. By contrast, those with low *veg-literacy*, little interest, or capacity to invest in vegetables or those constrained by the narrow range of vegetables their child will eat tend to only be interested in price promotions on the vegetables that they already buy, as a way of releasing money for other things. Health promotion programmes using supply-side interventions such as vouchers or price discounts intended to increase consumption of healthier foods may be effective for households with the capacity to respond, but for low-budget households, they may simply act as financial assistance through expenditure displacements rather than improving dietary intake.

### Practical implications

Most public health initiatives aiming to enable children and parents to buy and eat more vegetables, whether through media campaigns, price reductions, or choice architecture in supermarkets, are in effect asking parents to invest money, time, effort, and culinary confidence in preparing and cooking more vegetables. This ‘Ask’ has risks attached. For much of the general population, the risk may be negligible; households can readily absorb any waste or have levels of *veg-literacy* and confidence in their cooking skills to prevent it. But for parents who shop on a budget, our research suggests that limited *veg-literacy*, food norms, competing priorities, and complex lives mean that they had little motivation or opportunity to invest in what Bowen called ‘the invisible labour’ of incorporating more or different vegetables into family diets.^(49)^ Rather, shopping decisions are based on habit and familiar risk aversion strategies, which keep the household in balance.^(43)^

As noted by other authors, reducing economic barriers may not diminish dietary inequalities unless other barriers are addressed.^(23)^ The range of vegetables children are exposed to in childhood begins to set their social norms around diet and their emerging *veg-literacy* and is arguably a key part of their education for life, equipping them to enjoy the taste and culinary opportunities of a range of vegetables. Participants in our study valued school meals for introducing different vegetables and for the peer effect, which encouraged children to taste and experiment. Given the constraints facing low-income households, school meals could be a critical social leveller for exposure to vegetable variety and taste formation; mass catering is able to absorb, mitigate, and redeploy the inevitable food waste of children’s learning exposures to new vegetables.

The importance of *veg-literacy* and exposure to enhance vegetable familiarity and thus diversity and norms emerge as areas for potential programme development. This might require coordinated multisectoral interventions, for example, which align promotions in the supermarket for the general population with targeted measures that enable those with limited food budgets to access and taste the promoted vegetables cost free, perhaps via local meals services, food banks, or targeted give-aways via supermarket loyalty schemes. Project design needs to recognise that certain vegetables are intensely disliked and including them in promotions, mixed packs, or main ingredients for recipes or activities could limit uptake. Making vegetable promotions accessible to low-budget households has two components: strengthening parents’ capacity to respond (e.g. by increasing *veg-literacy*) and reducing the ‘ask’ by selecting vegetables that are easy to prepare. The potential of frozen vegetables and small pack sizes could be further explored.

Low vegetable intakes are a significant public health nutrition challenge globally.^(3)^ A recent review of food and health literacies noted the need for tighter alignment between health outcomes and the competencies required to achieve them.^(57)^ We propose that the concept of *veg-literacy* could be a valuable subset within food literacy for designing, setting objectives, and monitoring vegetable promotion interventions.

Since the research was carried out in 2018, the COVID-19 pandemic and subsequent global increases in the cost of living are likely to have intensified financial pressures on households with limited food budgets.^(58)^ The broad themes identified in our research remain pertinent; however, higher food prices will increase pressures for household food choices to be driven by children’s preferences as a risk aversion strategy. It will be important that school meals and community food offers are supported to counter any reduction in the diversity of vegetables used in the home due to households cutting costs. Our increasing awareness of the importance of diversity of fruit and vegetable consumption for gut health and the need to double global consumption of fruit and vegetables for planetary health to offset reductions in meat and dairy indicate that action to reduce inequalities in vegetable consumption remains relevant.^(14,56)^

### Strengths and limitations

The study’s strengths include its focus on vegetables and high representation of households from an area in the lowest quintile of IMD. The study provides depth, not breadth, and was conducted with a mostly white British ethnic group; understandings in other populations and places may differ. Participants self-selected to join the FGDs so might be more interested in food than the general population. We used photographs of vegetables as displayed in the freezer cabinet during the FGD rather than actual bags of frozen vegetables, and it is possible that this may have influenced participants’ readiness and comfort in talking about frozen vegetables.

### Conclusion

The present study suggests that without targeted measures directed towards population groups facing the biggest challenges, supply-side vegetable promotion programmes to the general population risk widening dietary inequalities. This applies to both short-term and long-term inequalities through the influence of vegetable exposure on shaping children’s taste acquisition and an intergenerational cycle of households where eating certain vegetables is not a household norm. We suggest that reducing the ‘Ask’ of eating more vegetables for low-budget households would entail promotions on vegetables, which can be eaten raw, have a good shelf life, and can be purchased in small quantities. Price promotions on ‘*core veg*’, which are already routinely bought, are more likely to result in expenditure displacement rather than increasing amounts or diversity of vegetables purchased.
